# Selective activation of pro-anti-IL-1β antibody enhances specificity for autoinflammatory disorder therapy

**DOI:** 10.1038/s41598-021-94298-y

**Published:** 2021-07-21

**Authors:** Wen-Wei Lin, Yun-Chi Lu, Bo-Cheng Huang, Chih-Hung Chuang, Yi-An Cheng, I.-Ju Chen, Hui-Ju Liu, Kai-Wen Ho, Tzu-Yi Liao, En-Shuo Liu, Ting-Yi Wu, Long-Sen Chang, Shih-Ting Hong, Tian-Lu Cheng

**Affiliations:** 1 Department of Laboratory Medicine, School of Medicine, College of Medicine, Kaohsiung Medical University, Kaohsiung, Taiwan; 2 Graduate Institute of Medicine, College of Medicine, Kaohsiung Medical University, 100 Shih-Chuan 1st Road, Kaohsiung, 80708 Taiwan; 3 Department of Biomedical and Environmental Biology, Kaohsiung Medical University, 100 Shih-Chuan 1st Road, Kaohsiung, 80708 Taiwan; 4 Drug Development and Value Creation Research Center, Kaohsiung Medical University, Kaohsiung, Taiwan; 5 Department of Medical Research, Kaohsiung Medical University Hospital, Kaohsiung, Taiwan; 6 Institute of Biomedical Sciences, National Sun Yat-Sen University, Kaohsiung, Taiwan; 7 Department of Medical Laboratory Science and Biotechnology, College of Health Sciences, Kaohsiung Medical University, Kaohsiung, Taiwan; 8 Department of Laboratory Medicine, Post Baccalaureate Medicine, College of Medicine, Kaohsiung Medical University, Kaohsiung, Taiwan

**Keywords:** Biological techniques, Biotechnology, Cancer

## Abstract

Canakinumab is a fully human monoclonal antibody that specifically neutralizes human interleukin (IL)-1β and has been approved by the US Food and Drug Administration for treating different types of autoinflammatory disorders such as cryopyrin-associated periodic syndrome, tumor necrosis factor receptor-associated periodic syndrome and systemic juvenile idiopathic arthritis. However, long-term systemic neutralization of IL-1β by Canakinumab may cause severe adverse events such as serious upper respiratory tract infections and inflammation, thereby decreasing the quality of life of patients. Here, we used an IgG1 hinge as an Ab lock to cover the IL-1β-binding site of Canakinumab by linking with matrix metalloprotease 9 (MMP-9) substrate to generate pro-Canakinumab that can be specifically activated in the inflamed regions in autoinflammatory diseases to enhance the selectivity and safety of treatment. The Ab lock significantly inhibited the IL-1β-binding by 68-fold compared with Canakinumab, and MMP-9 completely restored the IL-1β neutralizing ability of pro-Canakinumab within 60 min and blocked IL-1β-downstream signaling and IL-1β-regulated genes (i.e., IL-6). It is expected that MMP-9 cleavable and efficient Ab lock will be able to significantly enhance the selective reaction of Canakinumab at the disease site and reduce the on-target toxicities of Canakinumab during systemic circulation, thereby showing potential for development to improve the safety and quality of life of patients with autoinflammatory disorders in the future.

## Introduction

Interleukin (IL)-1β is a major pro-inflammatory cytokine that is involved in the initiation and persistence of inflammation and is essential for the defense of hosts against pathogenic infection and injury^1^. However, dysregulated IL-1β production by the innate immune system may cause abberant local or systemic inflammation and lead to different types of autoinflammatory disorders, such as cryopyrin-associated periodic syndrome (CAPS)^2^, tumor necrosis factor receptor associated periodic syndrome (TRAPS)^3^, systemic juvenile idiopathic arthritis (SJIA)^4^ or chronic obstructive pulmonary disease (COPD)^5^. IL-1β has been used as a target for developing therapeutic antibody (Ab) drugs. Canakinumab (Ilaris^®^) is a fully human monoclonal Ab that specifically neutralizes human IL-1β and has been approved by the US Food and Drug Administration (FDA) for treating CAPS^2^, TRAPS^3^ and SJIA^4^. It has also been evaluated for the inflammatory condition of COPD (Phase I clinical trial)^6,7^. However, systemic neutralization of IL-1β by Canakinumab may cause severe and undesirable adverse events, especially oppurtunistic infections. According to reports from the FDA and clinical trials, patients suffer nasopharyngitis (34% in CAPS and 10.7% in TRAPS), bronchitis (11% in CAPS), upper respiratory tract infection (7.1% in TRAPS) and pneumonia (2.4% in TRAPS and 4 to 5% in SJIA)^8^ after Canakinumab treatment, affecting the quality of life of both patients and informal caregivers^2,9,10^. Therefore, it is important to develop an anti-IL-1β Ab that is highly selective to the inflammatory region to prevent systemic on-target toxicities in Canakinumab-treated patients.

**Figure 1:**
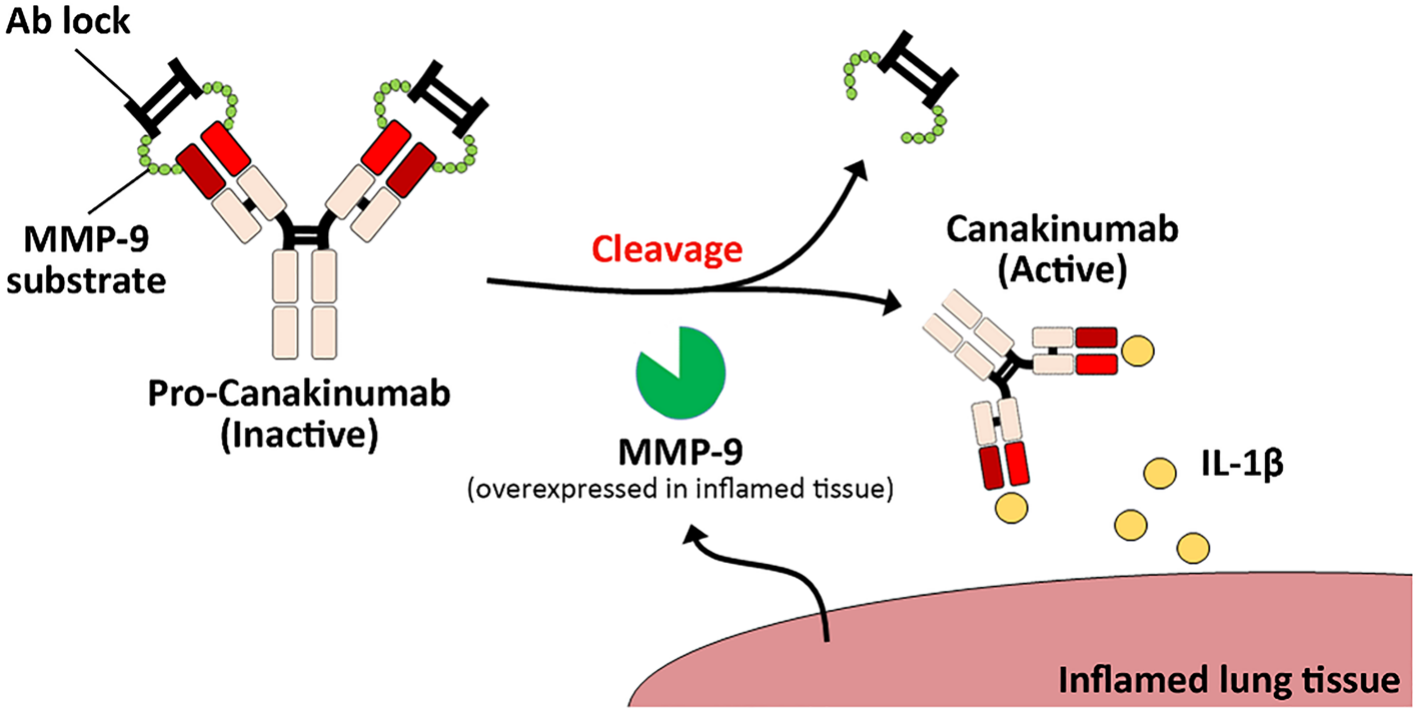
Schematic illustration of pro-Canakinumab selective activation by MMP-9 cleavage and specific neutralization of IL-1β at the inflamed region in autoinflammatory diseases. We engineered the human IgG1 hinge as an Ab lock (i.e. EPKSCDKTHTCPPCP) in front of the antigen-binding site of Canakinumab (fully human anti-human IL-1β mAb) by using MMP-9 substrate peptide as linker to generate pro-Canakinumab. After the Ab lock is removed by the MMP-9 expressed in the disease region, the cleaved pro-Canakinumab is expected to be specifically activated, neutralize the local IL-1β antigen and reduce the systemic on-target toxicity during autoinflammatory disease treatment.

In this study, we engineered a human IgG1 hinge as an Ab lock^11^ in front of the antigen-binding site of Canakinumab by using MMP-9 protease substrate as linker to generate pro-Canakinumab. After the Ab lock is removed by MMP-9, which is overexpressed in the disease region of autoinflammatory disorders such as CAPS^12^ or COPD^13–15^, the cleaved pro-Ab is expected to be specifically activated and neutralize the target antigen (Fig. 1). We first constructed and purified the pro-Canakinumab and confirmed the purity and molecular weight by sodium dodecyl sulfate (SDS)-polyacrylamide gel electrophoresis (PAGE). The masking efficiency and restoring ability of pro-Canakinumab after MMP-9 cleavage was analyzed by immunoassay. We further assessed the IL-1β neutralizing effect of pro-Canakinumab after treatment with MMP-9. Finally, we investigated the inhibitory effect of MMP-9-activated pro-Canakinumab to IL-1β downstream genes (i.e., IL-6) by Western blot and ELISA. It is expected that this universal Ab lock based on spatial-hindrance can effectively mask the antigen binding ability of Canakinumab, efficiently restore the biological activity by disease-associated proteases, and selectively react at the disease site to prevent severe on-target toxicities caused by systemic administration of Canakinumab.

## Materials and methods

### Cells and reagents

Human embryonic kidney cell line HEK293 and human lung carcinoma cell line A549 were purchased from American Type Culture Collection (USA) and cultured in Dulbecco’s modified Eagle’s medium (DMEM; Sigma-Aldrich, St Louis, MO, USA) supplemented with 10% (v/v) heat-inactivated bovine calf serum (BCS; Thermo Fisher Scientific, Waltham, MA, USA) and 100 units/mL penicillin and streptomycin (Invitrogen, Calsbad, CA) at 37 °C in a humidified atmosphere of 5% (v/v) CO_2_. The Expi293F cells (Thermo Fisher Scientific, Waltham, MA, USA) were cultured in Expi293 Expression Medium at 37 °C in a humidified atmosphere of 8% CO_2_. 2,2′-Azinobis[3-ethylbenzothiazoline-6-sulfonic acid] (ABTS) and 30% hydrogen peroxide were from Sigma-Aldrich. Horseradish peroxidase (HRP)-conjugated goat anti-human or -mouse IgG Fc and HRP-conjugated goat anti-mouse IgG F(ab′)_2_ secondary antibodies were from Jackson Immunoresearch Laboratories (West Grove, PA). Recombinant Interleukin (IL)-1β, IL-6 and human IL-6 DuoSet ELISA kit were purchased from R&D Systems (Minneapolis, MN, USA). Recombinant MMP-9 and bovine serum albumin (BSA) were purchased from Sigma-Aldrich (St. Louis, MO, USA).

### Plasmid construction, expression and purification

The complementary DNA coding for the heavy and light chains of Canakinumab were cloned through assembly PCR. Human IgG1 hinge sequences (i.e. EPKSCDKTHTCPPCP) were obtained from the National Center for Biotechnology Information (NCBI). The hinge-encoding sequences, GGGGS linker, and MMP-9 substrate-encoding sequences (GPLGVR)^16^ were introduced upstream of the light chain and heavy chain of Canakinumab to generate pro-Canakinumab. Canakinumab or pro-Canakinumab production were through the Expi293 Expression System (Thermo Fisher Scientific, Waltham, MA, USA) and purified by Protein A-Sepharose (GE Healthcare, Milwaukee, WI, USA).

### Western blot and SDS-PAGE

To examine the selective activation of pro-Canakinumab by MMP-9 treatment, 50 μg of Canakinumab and pro-Canakinumab were incubated with or without 50 μg/mL of MMP-9 for 0, 15, 30 or 60 min, respectively. All samples were mixed with 6 × SDS reducing loading dye and boiled for 10 min. Samples were separated by 10% SDS-PAGE and then transferred to nitrocellulose (NC) membranes (Millipore, Billerica, MA, USA). After blocking with phosphate-buffered saline (PBS) containing 5% milk at 4 °C overnight, the membranes were incubated with HRP-conjugated goat anti-human IgG F(ab′)_2_ secondary antibodies (Jackson ImmunoResearch Laboratories, West Grove, PA, USA) at room temperature (RT) for 1 h. After extensive washing, the blots were visualized by enhanced chemiluminescence detection according to the manufacturer’s instructions (Merck Millipore). To analyze the molecular weight (MW) and purity of purified Canakinumab and pro-Canakinumab, 2 μg of each antibody was separated by 10% SDS-PAGE and then stained with Coomassie Brilliant Blue.

### Enzyme-linked immunosorbent assay

To determine the binding kinetics (half-maximal effective concentration; EC_50_) of pro-Canakinumab and Canakinumab, 50 μL recombinant IL-1β (0.3 μg/mL) was coated onto 96-well plates and blocked with 5% skim milk in PBS. 500 nM Canakinumab or pro-Canakinumab was incubated with or without 100 μg/mL of MMP-9 in DMEM containing 0.05% BSA (pH: 7.4) for 1 h at 37 °C before the reaction was terminated by 25 μL BCS. All the samples were added 50 μL onto the plates at the given concentrations (0.128–400 nM) for 1 h at RT. After washing, the wells were incubated with 50 μL HRP-goat anti-human IgG Fc secondary antibody for 1 h at RT, and detection was performed by the addition of 150 μL ABTS solution [0.4 mg/mL, 2,2′-Azinobis[3-ethylbenzothiazoline-6-sulfonic acid] (Sigma-Aldrich), 0.01% (v/v) H_2_O_2_, and 100 mM phosphate-citrate, pH 4.0]. Color development was measured at 405 nm on a microplate reader (Molecular Devices, Menlo Park, CA, USA).

The expression level of IL-6 was detected by human IL-6 DuoSet ELISA kit (R&D Systems, Minneapolis, MN, USA) according to the manufacturer’s protocol. Briefly, 1.2 × 10^5^ human lung carcinoma cell line A549 was seeded into 24-well plates overnight in an incubator at 37 °C. After starving cells with serum free medium for 24 h, the medium was replaced with IL-1β (5 ng/mL, R&D Systems), and incubated with saline, Canakinumab (2 nM or 10 nM), pro-Canakinumab (2 nM or 10 nM), 100 μg/mL MMP-9 pre-incubated Canakinumab or 100 μg/mL MMP-9 pre-incubated pro-Canakinumab for 24 h at 37 °C. Supernatant of each of the treated groups (n = 3) was added onto 2 μg/mL of IL-6 capture antibody-coated 96-well plates at RT for 2 h after the coated plates were blocked with 300 μL 1% BSA in PBS. After washing, the wells were sequentially incubated with 50 ng/mL of biotinylated IL-6 detection antibody and HRP-conjugated streptavidin. The plates were washed with PBST (PBS containing 0.05% Tween 20) and bound peroxidase activity was measured by adding 100 μL substrate solution into each well for 20 min at RT; the reaction was stopped by adding 50 μL stop solution. Color development was measured at 450 nm and 570 nm on a microplate reader.

### Luciferase reporter assay

Human embryonic kidney cell line (HEK293) cells were transiently transfected with the luciferase reporter plasmid pNF-κB-Luc^17^ (BD Biosciences Clontech, Palo Alto, CA, USA), internal control Renilla-Luc reporter plasmid with PureFection reagent (System Biosciences, Palo Alto, CA, USA) for 6 h. After the transfection procedure, the medium was replaced with IL-1β (10 ng/mL, R&D Systems), and incubated with 50 nM Canakinumab, 50 nM pro-Canakinumab, 9.6 μg MMP-9 pre-incubated Canakinumab or 9.6 μg MMP-9 pre-incubated pro-Canakinumab in 1 mL serum free medium for 1 h at 37 °C. After removal from the medium, the cells were cultured for 16 h at 37 °C, cell extracts from each sample were measured using the dual-luciferase reporter assay system (Promega, Madison, WI, USA) according to the manufacturer’s protocol.

### Statistical analysis

Data are presented as mean ± SD. All the readings were background adjusted by subtracting the absorbance of a blank control in the ELISA procedures. The expression level of IL-6 was determined by using an independent *t* test to compare the statistical significance of the differences between the controls and samples. Statistical analysis was performed using the GraphPad Prism v.6 and Data were considered significant at a *p* value of < 0.05.

## Results

### Characterization of pro-Canakinumab

**Figure 2:**
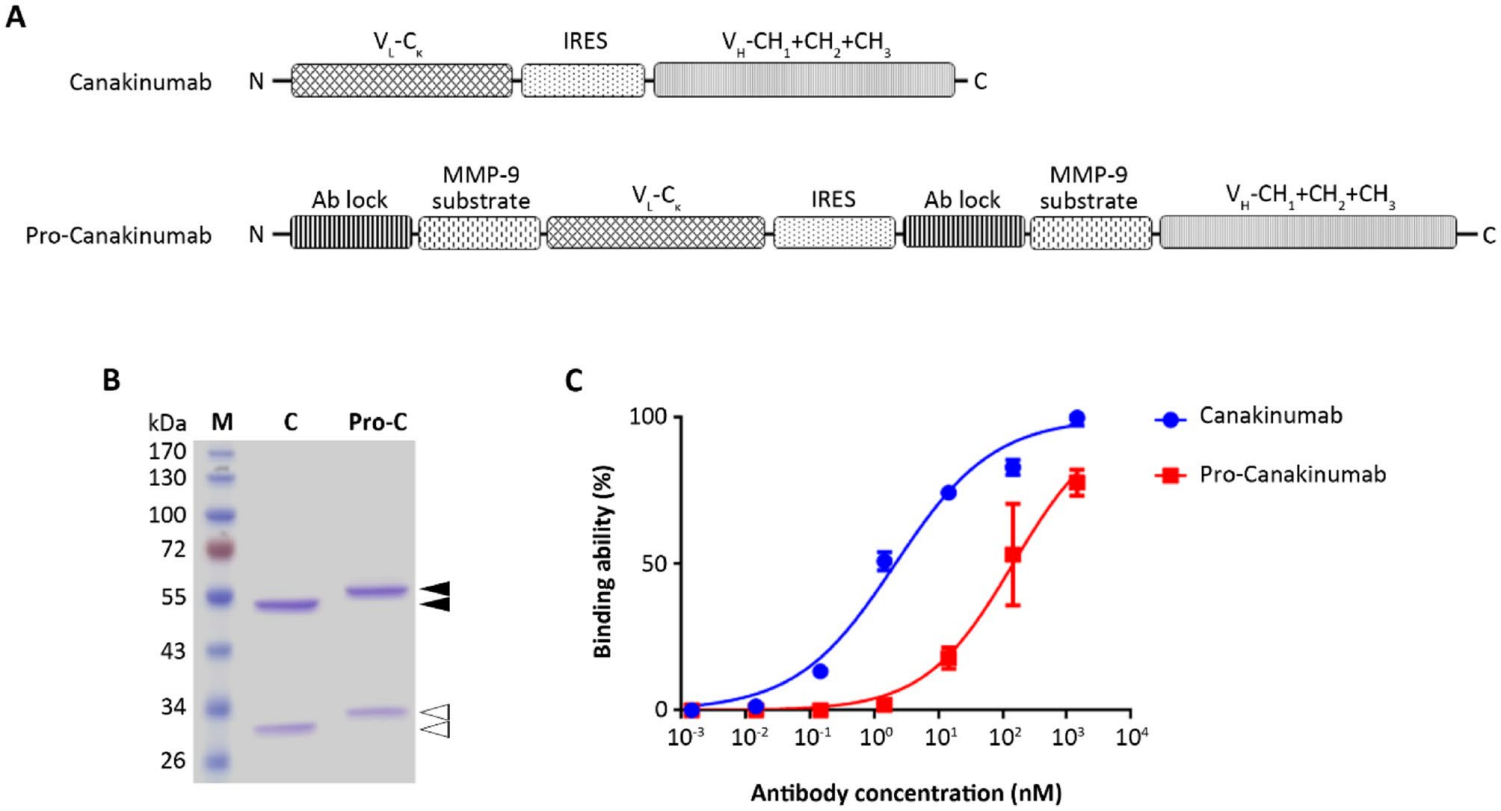
Generation and characterization of pro-Canakinumab. (**A**) Schematic of the Canakinumab (fully human anti-human IL-1β mAb) including (from the N to C terminus) the light chain (V_L_-C_κ_) and heavy chain (V_H_-CH1 + CH2 + CH3) of Canakinumab that link with an internal ribosomal entry site (IRES). The pro-Canakinumab includes (from the N to C terminus) the IgG1 hinge (Ab lock) in front of the light chain (V_L_-C_κ_) and heavy chain (V_H_-CH1 + CH2 + CH3) of Canakinumab, respectively, and these two fragments are also joined by an IRES. (**B**) SDS-PAGE analysis of protein A-purified Canakinumab and pro-Canakinumab: lane 1, molecular weight marker; lane 2, Canakinumab; lane 3, pro-Canakinumab. The black arrow indicates the heavy chain fragments of Canakinumab or pro-Canakinumab, respectively. The white arrow indicates the light chain fragments of Canakinumab or pro-Canakinumab, respectively. (**C**) The binding ability of Canakinumab (blue circles) and pro-Canakinumab (red squares) was assessed by IL-1β-based ELISA. The percentage of mean absorbance values (405 nm) of triplicate determinations are shown (n = 3). The bars indicate the SD.

To evaluate the masking efficiency of the Ab lock (IgG1 hinge domain) to Canakinumab, the Ab lock was linked to the N-terminus of light chain and heavy chain of Canakinumab, respectively, through a MMP-9 substrate linker (GVLGVR) to generate pro-Canakinumab. MMP-9 is overexpressed in the inflamed region of CAPS^12^ and COPD^13–15^ and is suitable to serve as an unlocked protease in these autoinflammatory disorders. The engineered light chain and heavy chain were then linked through internal ribosome entry site (IRES) (Fig. 2A), which allows the simultaneous expression of two polypeptides separately, but from the same RNA transcript^18,19^, and cloned to mammalian-expressing vectors pLJCX to form pLJCX-pro-Canakinumab expression vector. After large scale production of pro-Canakinumab and Canakinumab by the Expi293 expression system and purification by a protein A column, we confirmed the molecular weight and purity of purified antibodies by SDS-PAGE. Figure 2B shows that the heavy chains and light chains of pro-Canakinumab and Canakinumab had apparent molecular weights of approximately 58 kDa, 29.4 kDa, 54 kDa and 26.3 kDa, respectively, consistent with their expected molecular weights (Fig. 2B). And the purity of purified pro-Canakinumab and Canakinumab both achieved 95% (Fig. 2B). We further investigated the masking effect of the Ab lock by comparing the IL-1β binding ability of pro-Canakinumab and Canakinumab by ELISA. The EC_50_ of pro-Canakinumab and Canakinumab were 2.103 nM and 143.7 nM, respectively, showing that pro-Canakinumab has an approximately 68-fold weaker antigen binding ability than Canakinumab (Fig. 2C). These results suggest that we have successfully engineered pro-Canakinumab, which can efficiently be blocked by the Ab lock and significantly attenuated its antigen binding ability in comparison with the original Canakinumab.

### Restoration of IL-1β binding ability of pro-Canakinumab after protease cleavage

**Figure 3:**
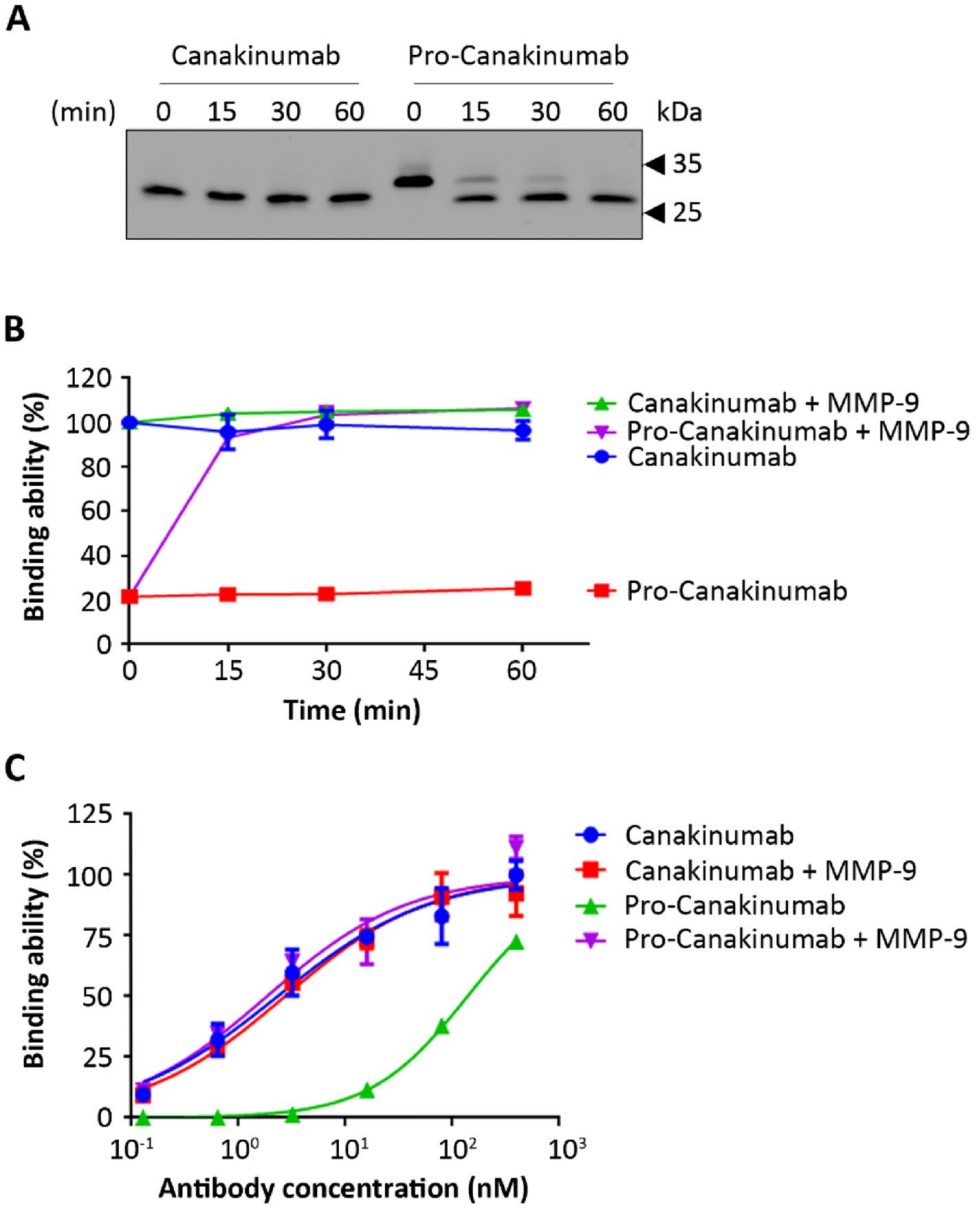
Selective activation of pro-Canakinumab by MMP-9 treatment. Canakinumab or pro-Canakinumab (25 nM) were incubated with 4 μg recombinant MMP-9 for 0, 15, 30 and 60 min, respectively. (**A**) The light chain molecular weight of the Canakinumab or active and inactive pro-Canakinumab were detected with HRP-conjugated anti-human IgG Fab Ab by Western blot and (**B**) the IL-1β binding ability of Canakinumab and pro-Canakinumab with or without MMP-9 treatment were analyzed by IL-1β-coated ELISA (n = 3). (**C**) The IL-1β antigen coated 96-well plate was also incubated with different concentrations of Canakinumab (blue circles), pro-Canakinumab (green upward triangle), Canakinumab pre-incubated with MMP-9 (red squares) or pro-Canakinumab pre-incubated with MMP-9 (pink downward triangles), respectively, and then the IL-1β binding ability was analyzed by ELISA. The percentage of mean absorbance values (405 nm) of triplicate determinations are shown (n = 3). The bars indicate the SD.

To investigate whether the Ab lock can be efficiently removed from pro-Canakinumab and restore the antigen binding ability of pro-Canakinumab after MMP-9 cleavage, we incubated pro-Canakinumab and Canakinumab with MMP-9 for different amounts of time and evaluated the molecular weight profile of the antibody fragments by Western blot. The results show that the light chain (29.4-kDa) molecular weight of pro-Canakinumab was converted into the profile similar to the control Canakinumab (25.6-kDa for light chain) after treatment with MMP-9 for under 60 min (Fig. 3A), demonstrating that the MMP-9 could completely remove the Ab lock from pro-Canakinumab. The antigen binding ability was also analyzed by ELISA. As shown in Fig. 3B, the IL-1β binding ability of pro-Canakinumab was gradually elevated in a time-dependent manner during MMP-9 treatment and finally restored to a level similar to the control Canakinumab. The EC_50_ of Canakinumab and pro-Canakinumab were 2.321 nM and 139 nM, respectively, showing that pro-Canakinumab had an approximately 60-fold weaker antigen binding ability than Canakinumab (Fig. 3C). After reacting with MMP-9 protease, the EC_50_ of pro-Canakinumab was elevated from 139 to 1.848 nM and showed no significant difference (*p* = 0.8) compared with the control Canakinumab (Fig. 3C). We further investigated that whether the antigen binding ability of pro-Canakinumab can be restored in physiological MMP concentration of inflamed region. We incubated pro-Canakinumab or Canakinumab with MMP from 2.7 to 75 ng/mL, which is below or in the range of MMP-2 (37 ± 2.8 ng/mL) or MMP-9 (1.7 ± 0.25 μg/mL) expression level in the joint fluid of rheumatoid arthritis (RA) patients^20^, for 1 h and detected the IL-1β-binding ability by ELISA. Result shows that the IL-1β binding ability of pro-Canakinumab can be gradually restored after incubating with different physiological concentration of MMP and can be completely recovered at MMP concentration by 8.3 ng/mL (Supplementary Figure S1), indicating that physiological concentration of MMP is sufficient to activate the pro-Ab in inflamed region. In order to analyze that whether pro-Canakinumab can be selectively activated in vivo, we intraperitoneally treated 50 μg pro-Canakinumab to wild-type (WT) mice (i.e. DBA/1 mice) or inflammatory mice model (i.e. collagen-induced arthritis (CIA) mice model, which will express high level MMP-2 and MMP-9 in the inflamed paw. We collected the blood samples at different time points (0, 6 and 24 h after treatment) and inflammatory tissues (paw) and normal lung organ at the end point of the experiment, then detected the expression level of active and inactive pro-Canakinumab by Western blot. Results indicated that the heavy chain molecular weight (58 kDa) of pro-Canakinumab was only converted into cleaved form (54 kDa) in the paw of CIA model, but not in the normal lung organ of CIA model or in the lung and paw tissue of WT mice at 24 h after treatment (Supplementary Figure S2). Nevertheless, the heavy chain molecular weight (58 kDa) in the serum of pro-Canakinumab-treated WT mice and CIA mice model were still maintained in uncleaved form until 24 h after treatment (Supplementary Figure S3). These results indicate that the Ab lock can be efficiently removed from the antigen binding site of pro-Canakinumab and completely restore the IL-1β binding ability of pro-Canakinumab after cleavage by physiological concentration of MMP-9.

### IL-1β-neutralizing ability of pro-Canakinumab after protease cleavage

**Figure 4:**
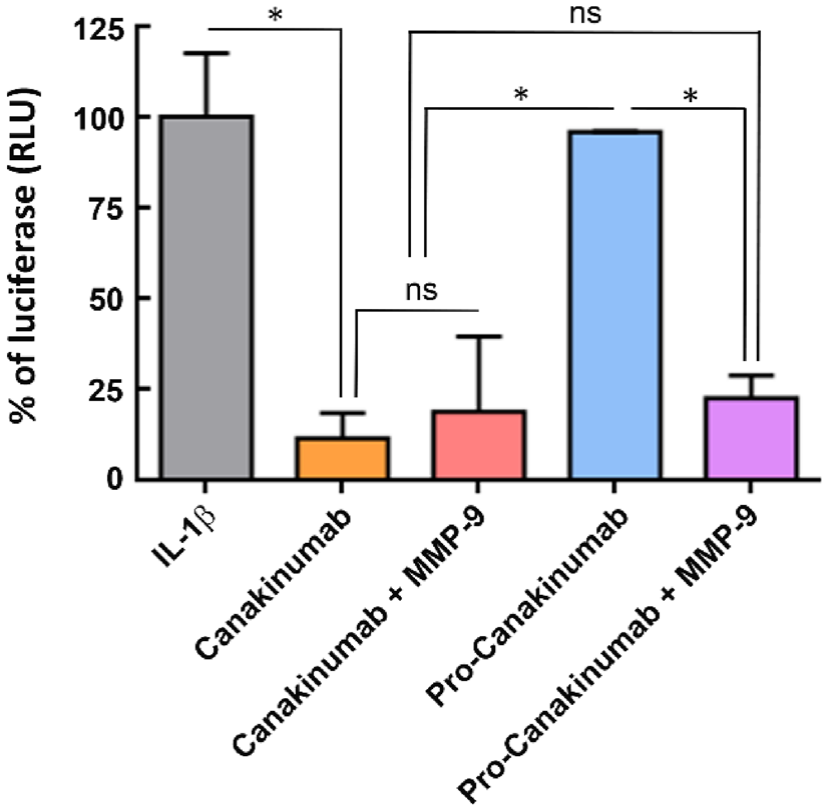
The neutralizing effect of pro-Canakinumab on IL-1β downstream signaling. Luciferase activity of NF-κB promoter constructs (pNF-κB-Luc) transiently transfected into HEK293 cells with or without treatment with IL-1β (10 ng/mL). IL-1β neutralizing ability was analyzed by incubating cells with saline (gray), Canakinumab (orange), pro-Canakinumab (blue), MMP-9 pre-incubated Canakinumab (pink) or MMP-9 pre-incubated pro-Canakinumab (purple) and the luciferase activity was detected through a dual-luciferase reporter assay system (Promega, Madison, WI, USA) according to the manufacturer’s protocol. IL-1β treatment was used as a control for reporter activity. The results are expressed as fold changes of luciferase activity (relative light unit, RLU) over those of internal control Renilla–Luc reporter plasmid. All data are the mean ± SD of triplicate independent experiments (n = 3). Statistical significance was calculated by *t* test with NS, no significance; *P < 0.05.

**Figure 5:**
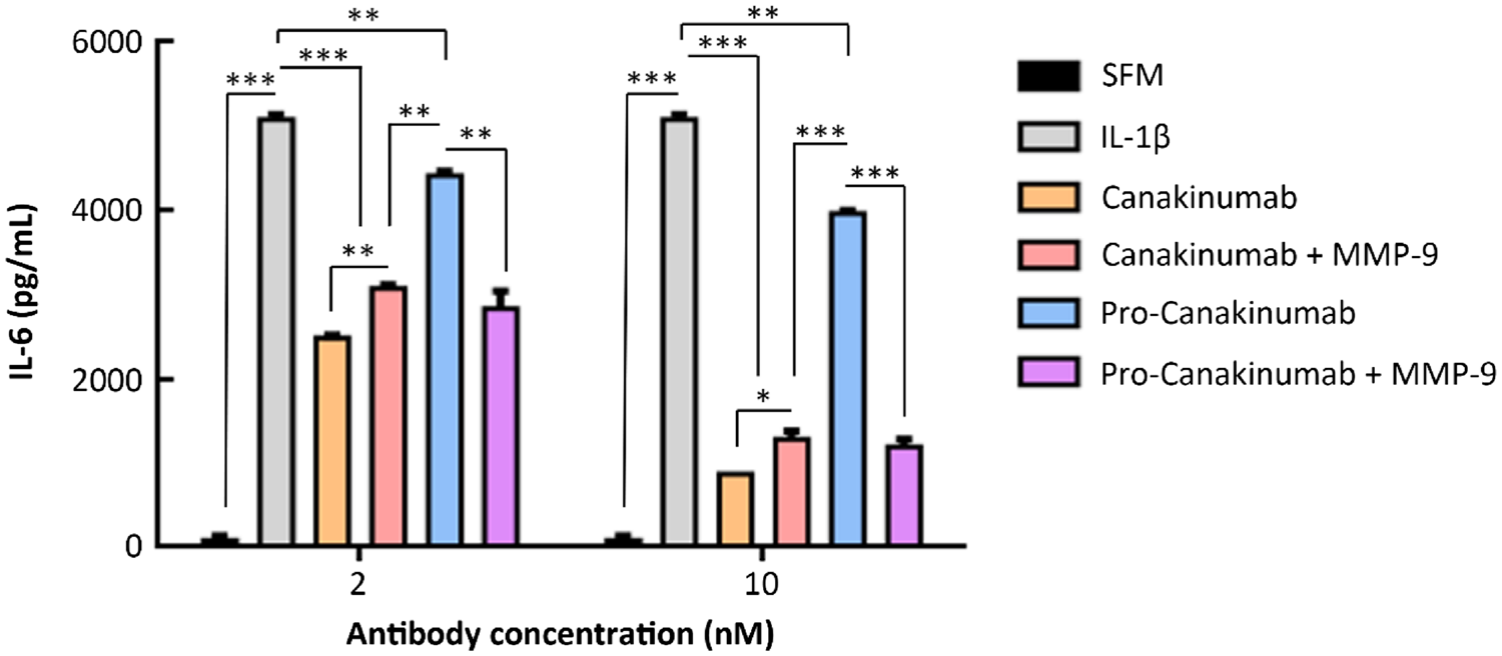
Effect of pro-Canakinumab on IL-1β-induced IL-6 expression. Human lung carcinoma cell line A549 (1.2 × 10^5^) was treated with IL-1β (5 ng/mL, gray), and incubated with serum free medium (SFM) (black), Canakinumab (orange), pro-Canakinumab (blue), MMP-9 pre-incubated Canakinumab (pink) or MMP-9 pre-incubated pro-Canakinumab (purple) for 24 h. The supernatant was collected and the expression level of IL-6 was detected by human IL-6 DuoSet ELISA kit (R&D Systems, Minneapolis, MN, USA) according to the manufacturer’s protocol. All data are the mean ± SD of triplicate independent experiments (n = 3). Statistical significance was calculated by *t* test with *P < 0.05; **P < 0.01; ***P < 0.001 when compared between each indicated group.

We next analyzed the IL-1β-neutralizing ability of MMP-9 activated pro-Canakinumab by nuclear factor kappa B (NF-κB) reporter assay^21^. We co-incubated recombinant IL-1β with Canakinumab, pro-Canakinumab, MMP-9 pre-incubated Canakinumab or MMP-9 pre-incubated pro-Canakinumab to pNF-κB-Luc- and Renilla-Luc control plasmid-co-transfected HEK293 cells followed by monitoring the luciferase activity by using dual-luciferase reporter assay system. Figure 4 shows that the luciferase activity of pro-Canakinumab-treated cells was similar to the IL-1β-treated group (*p* = 0.7714). In contrast, MMP-9-activated pro-Canakinumab could significantly neutralize IL-1β and reduce the IL-1β-induced luciferase activity similar to the Canakinumab-treated group (*p* = 0.236) (Fig. 4), suggesting that the pro-Canakinumab could efficiently neutralize IL-1β and block the downstream NF-κB signaling after MMP-9 cleavage. We further investigated whether MMP-9-activated pro-Canakinumab can inhibit the expression of IL-1β downstream-related genes. The expression level of IL-6 was measured by IL-6 sandwich ELISA after treating human lung carcinoma cell line A549 with IL-1β and Canakinumab, pro-Canakinumab, MMP-9 pre-incubated Canakinumab or MMP-9 pre-incubated pro-Canakinumab, respectively. Figure 5 shows that pro-Canakinumab significantly inhibits IL-1β-induced IL-6 expression after MMP-9 treatment as compared with the pro-Canakinumab group (*p* < 0.001) in a dose-dependent manner. In contrast, there was no significant difference in IL-6 expression observed between the MMP-9 pre-incubated Canakinumab and the MMP-9 pre-incubated pro-Canakinumab group (*p* = 0.2591 for 2 nM and *p* = 0.4389 for 10 nM antibody concentration). Taken together, these results suggest that the Ab lock markedly masks the IL-1β neutralizing ability of Canakinumab and the restricted biological function of pro-Canakinumab was completely reversed after MMP-9 cleavage.

## Discussion

We have successfully developed protease selective activated pro-Canakinumab using a previously developed autologous hinge domain, which can efficiently mask the IL-1β binding ability of Canakinumab. This pro-Canakinumab is selectively activated by autoinflammatory disease-associated MMP-9, and shows similar antigen binding ability to the original Canakinumab. We also proved that the MMP-9-activated pro-Canakinumab can further neutralize the IL-1β stimulation and impair IL-1β-induced NF-κB signaling and its downstream regulated gene expression (i.e., IL-6). We believe that the MMP-9 cleavable and efficient Ab lock can significantly enhance the selective reaction of Canakinumab at the disease site and reduce the on-target toxicities of Canakinumab during systemic circulation, thereby having potential for development to improve the safety and quality of life of patients with autoinflammatory disorders in the future.

The spatial-hindrance-based Ab lock is suitable for developing protease-activated pro-Abs. Unlike affinity-based masking peptides^11,22–24^, the masking effect of the Ab lock based on spatial-hindrance is easier recover after protease cleavage. Our results showed that the light chain (29.4-kDa) molecular weight of pro-Canakinumab was converted into a profile similar to the control Canakinumab (25.6-kDa for light chain) after treatment with MMP-9 for 60 min (Fig. 3A). Moreover, the EC_50_ of pro-Canakinumab was elevated from 139 to 1.848 nM after MMP-9 treatment and showed no significant difference (*p* = 0.8) compared with control Canakinumab (Fig. 3C). These results indicate that the spatial-hindrance-based Ab lock can be efficiently removed from the antigen binding site of pro-Canakinumab and completely restore the IL-1β binding ability of pro-Canakinumab after MMP-9 cleavage. Our previous study showed that the spatial-hindrance-based Ab lock can significantly prevent the neutralizing effect of the anti-idiotypic (Id) Abs to Infliximab, which is one of the major issues in rheumatoid arthritis (RA) therapy^25^. In addition, due to the highly conserved sequence and structure of human Abs, the spatial-hindrance-based Ab lock has more chance to widely mask the antigen binding ability of various Ab drugs in clinical use or in pre-clinical studies. We expect that this spatial-hindrance-based Ab lock may provide a better option for developing pro-Ab drugs which need high masking efficacy and strong restoration properties.

It is important to selectively activate the anti-IL-1β Ab in the inflamed regions of autoinflammatory diseases to prevent undesired immune-related adverse events (irAE) during treatment. Several complications accompany the symptoms of autoinflammatory diseases, for example, muscular weakness^26^, heart failure^27^ or pneumonia^28^ accompany COPD symptoms. However, the main therapeutic target of COPD, IL-1β, is a major cytokine involved in the initiation and persistence of inflammation. Long-term systemic neutralization of IL-1β by Canakinumab may increase the risk of serious upper respiratory tract infections, gastrointestinal disorders and vertigo, and decrease the quality of life of both patients and informal caregivers^2,9,10,29^. Thus, it is important to minimize the irAE of anti-IL-1β therapy. In our study, we developed a pro-Canakinumab, which can be selectively reactivated at the inflammatory site of RA that expresses high levels of MMP-9 (Supplementary Figure S2), and may locally and specifically neutralize IL-1β in the disease region, thereby preventing systemic on-target toxicities. In our previous study, we generated a protease-cleavable pro-anti-TNFα Ab (i.e. pro-Infliximab) and proved that the pro-Infliximab can not only selectively be activated by MMP protease at diseases region, provide equivalent therapeutic efficacy to Infliximab but also maintain mouse immunity against *Listeria* infection in the RA mice model, leading to a significantly higher survival rate (71%) than that of the Infliximab treatment group (0%)^25^. On the other hand, as an intact antibody, our Ab lock (i.e., the hinge domain) is one of the most abundant serum proteins in human blood. The immune system should tolerate self-antigens and prevent Hinge-induced immune responses. There may be concern that the administration of the pro-Ab could induce the production of anti-hinge Abs. We previously analyzed the immunogenicity of a pro-Ab (i.e., pro-Infliximab) by treating differentiated primary dendritic cells that were co-cultured with CD4^+^ T cells with synthetic MMP-2/9 substrate sequence, Infliximab or pro-Infliximab, respectively. The result showed that there was no significant difference in CD4^+^ T cell proliferation between MMP-2/9 substrate sequence-treated, Infliximab-treated, or pro-Infliximab-treated groups, and the control group^25^. Together, we expect that pro-Canakinumab is a low-immunogenic Ab drug that can be selectively restored and locally neutralize IL-1β at the disease region after MMP-9 treatment. This treatment may reduce the cost and undesirable side effects during long-term therapy in autoinflammtory diseases (e.g., COPD).

The flexible pro-Ab design strategy is important to widely confer the selectivity of disease region to different monoclonal Abs (mAbs) against different disease-associated target antigens. Several Ab drugs have been approved by the FDA or investigated in clinical trials for treating different autoinflammatory diseases. For example, Cocho et al. administrated Adalimumab (fully human anti-TNFα mAb) to a 7-year-old boy with TRAPS-associated panuveitis and efficiently inhibited the progression of existing lesions appeared for at least 16 months^30^. Benedetti’s group treated Tocilizumab (humanized anti-IL-6 receptor mAb) to 112 children with SJIA and significantly improved the 80% response rate in the Tocilizumab-treated group as compared with a placebo group after treatment with Tocilizumab for 52 weeks^31^. In addition, due to the limited benefit provided by current therapies for COPD, several potential therapeutic targets, such as COPD-associated proinflammatory cytokines^32^ and proteases^33^, are being investigated. For instance, a subset of COPD patients have elevated IL-5 levels in their blood and airways^34,35^ and there are two monoclonal Ab (mAb) drugs (i.e., Mepolizumab and Benralizumab) that target to IL-5 or IL-5 receptor alpha (IL-5Rα), respectively, that have been investigated for their ability to reduce exacerbation rate in COPD patients (NCT02105948, NCT01463644, NCT02105961 and NCT01227278). Lee and colleagues suggested that the overexpressed IL-13 may induce goblet cell hyperplasia, mucus hypersecretion and negatively correlated with the forced expiratory volume in 1 s (FEV_1_) of COPD patients^36–38^ and a 24-week treatment study of Lebrikizumab, which is a humanized anti-IL-13 mAb, for decline in frequency of COPD exacerbations and lung function is ongoing (NCT02546700). Lee et al. also indicated that the expression of MMP-13 was increased in lung tissues of COPD patients by MALDI-TOF MS analysis, which is a major proteolytic enzyme believed to be involved in tissue damage and remodeling^39^. Based on the variety of therapeutic target antigens and overexpressed proteases, our flexible design of protease substrates or therapeutic Abs in the pro-Ab can be widely and simply applied to different therapeutic strategies for different autoinflammatory disorders.

## Conclusion

In conclusion, we developed a protease-activated pro-Canakinumab in an attempt to attain a more efficient and safe treatment for autoinflammatory diseases by enhancing the selectivity of Ab drugs to the inflamed disease region. The pro-Ab drug has the following advantages: (1) it possess a high masking effect to IL-1β antigen under the region without inflammation and efficient restoration of IL-1β-neutralizing ability after protease cleavage; (2) the spatial-hindrance-based Ab lock and the flexible design of pro-Abs can be broadly applied to any disease-associated proteases or Ab drugs that are used to treat different autoinflammatory diseases; (3) it may represent the lowest immunogenic masking domain, because Abs are one of the most abundant protein components in human serum. We expect that this novel pro-Canakinumab can significantly enhance the selective reaction of Canakinumab at the disease site and reduce the on-target toxicities of Canakinumab during systemic circulation, thereby showing potential to improve the safety and quality of life of patients with autoinflammatory disorders in the future.

## Supplementary Information

Supplementary Figures.
