# Supplementary material for: Selective activation of pro-anti-IL-1β antibody enhances specificity for autoinflammatory disorder therapy
Source: Sci Rep. 2021 Jul 21;11:14846. doi: 10.1038/s41598-021-94298-y (PMC8295355; doi:10.1038/s41598-021-94298-y)
Supplement: Supplementary file 1 — Supplementary Figures. [file 41598_2021_94298_MOESM1_ESM.docx]

**Selective activation of pro-anti-IL-1β antibody enhances specificity for autoinflammatory disorder therapy**

Wen-Wei Lin^1,2,4,5,8^, Yun-Chi Lu^3,4^, Bo-Cheng Huang^6^, Chih-Hung Chuang^4,7^, Yi-An Cheng^3,4^, I-Ju Chen^3,4^, Hui-Ju Liu^2^, Kai-Wen Ho^2^, Tzu-Yi Liao^2^, En-Shuo Liu^4^, Ting-Yi Wu^2,4^, Long-Sen Chang^6^, Shih-Ting Hong^2,*^, Tian-Lu Cheng^2,3,4,5,6,**^

^1^Department of Laboratory Medicine, School of Medicine, College of Medicine, Kaohsiung Medical University, Kaohsiung, Taiwan

^2^Graduate Institute of Medicine, College of Medicine, Kaohsiung Medical University, Kaohsiung, Taiwan

^3^Department of Biomedical Science and Environmental Biology, Kaohsiung Medical University, Kaohsiung, Taiwan

^4^Drug Development and Value Creation Research Center, Kaohsiung Medical University, Kaohsiung, Taiwan

^5^Department of Medical Research, Kaohsiung Medical University Hospital, Kaohsiung, Taiwan

^6^Institute of Biomedical Sciences, National Sun Yat-Sen University, Kaohsiung, Taiwan

^7^Department of Medical Laboratory Science and Biotechnology, College of Health Sciences, Kaohsiung Medical University, Kaohsiung, Taiwan

^8^Department of Laboratory Medicine, Post Baccalaureate Medicine, College of Medicine, Kaohsiung Medical University, Kaohsiung, Taiwan

^*^Correspondence: Graduate Institute of Medicine, College of Medicine, Kaohsiung Medical University, 100 Shih-Chuan 1st Road, Kaohsiung, 80708 Taiwan. Tel.: +886 7 3121101 2697. Fax: +886 7 3227508. E-mail: gwu714@gmail.com

^**^Correspondence: Department of Biomedical and Environmental Biology, Kaohsiung Medical University, 100 Shih-Chuan 1st Road, Kaohsiung, 80708 Taiwan. Tel.: +886 7 3121101 2697. Fax: +886 7 3227508. E-mail: tlcheng@kmu.edu.tw

**Supplementary Information**

**Materials and Methods**

**Enzyme-Linked Immunosorbent Assay**

To determine the restoring efficiency of pro-Canakinumab after cleavaged with physiological concentration of MMP, 50 μL recombinant IL-1β (0.3 μg/mL) was coated onto 96-well plates and blocked with 5% skim milk in PBS. 63 nM Canakinumab or pro-Canakinumab was incubated with 0, 2.7, 8.3, 25, 75 ng/mL of pure MMP in DMEM containing 0.05% BSA (pH: 7.4) for 1 h at 37°C before the reaction was terminated by 25 μL BCS. All the samples were added 50 μL onto the IL-1β-coated plates for 1 h at RT. After washing, the wells were incubated with 50 μL HRP-goat anti-human IgG Fc secondary antibody for 1 h at RT, and detection was performed by the addition of 150 μL ABTS solution [0.4 mg/mL, 2,2′-Azinobis[3-ethylbenzothiazoline-6-sulfonic acid] (Sigma-Aldrich), 0.01% (v/v) H_2_O_2_, and 100 mM phosphate-citrate, pH 4.0]. Color development was measured at 405 nm on a microplate reader (Molecular Devices, Menlo Park, CA).

**Western Blot**

To examine the selective activation of pro-Canakinumab by MMP-9 treatment, 50 μg of pro-Canakinumab was intraperitoneally injected into DBA/1 mice (WT mice) or CIA mice model^1^, and collected their blood samples at different time points (0, 6 and 24 h after treatment) and inflammatory tissues (paw) and normal lung organ at the end point of the experiment. The protein lysate of tissues were extracted by RIPA lysis buffer and quantified by BCA assay. 32 μL serum samples or 30 μg total protein from each tissues were mixed with 6× SDS reducing loading dye and boiled for 10 min. Samples were separated by 10% SDS-PAGE and then transferred to nitrocellulose (NC) membranes (Millipore, Billerica, MA, USA). After blocking with phosphate-buffered saline (PBS) containing 5% milk at 4°C overnight, the membranes were incubated with rabbit anti-mouse β-actin, HRP-conjugated goat anti-rabbit IgG Fc secondary antibodies or HRP-conjugated goat anti-human IgG Fc secondary antibodies (Jackson ImmunoResearch Laboratories, West Grove, PA, USA) at room temperature (RT) for 1 h. After extensive washing, the blots were visualized by enhanced chemiluminescence detection according to the manufacturer’s instructions (Merck Millipore).


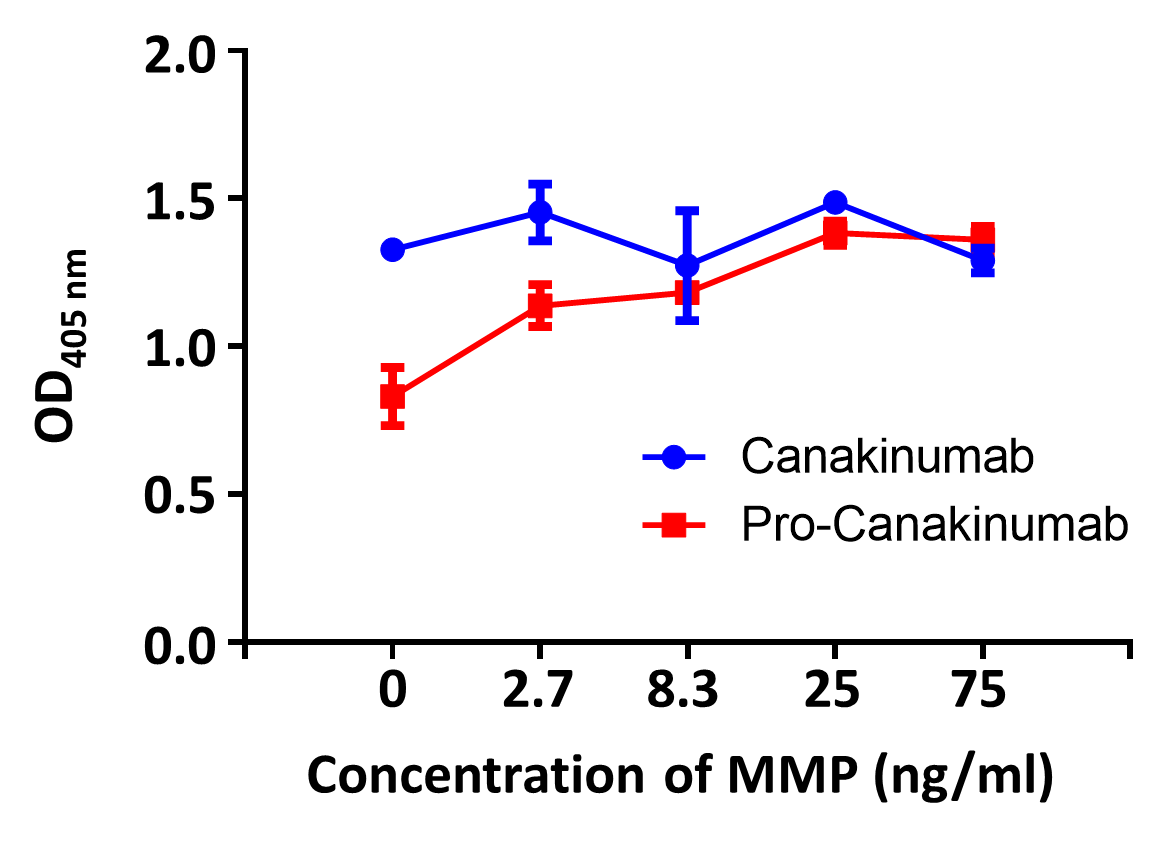


**Supplementary Figure S1: Selective activation of pro-Canakinumab by physiological concentration of MMP-9 treatment.** Canakinumab or pro-Canakinumab (63 nM) were incubated with different concentration of recombinant MMP-9 (0, 2.7, 8.3, 25, 75 ng/mL) for 1 h, respectively. The IL-1β binding ability of Canakinumab (●) and pro-Canakinumab (■) with or without MMP-9 treatment were analyzed by IL-1β-coated ELISA. The mean absorbance values (405 nm) of triplicate determinations are shown (n=3). The bars indicate the S.D.


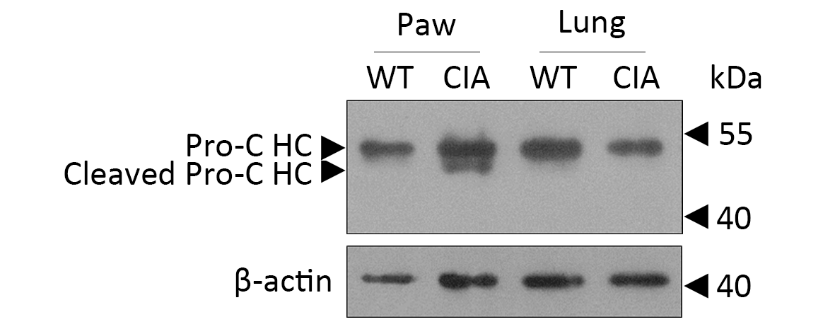


**Supplementary Figure S2. The pro-Canakinumab is specifically activated at the inflamed site.** We treated 50 μg pro-Canakinumab to wild-type (WT) mice or collagen-induced arthritis (CIA) mice model, respectively, collected their joint tissues (paw) and normal lung organ and using anti-β-actin Ab, HRP-conjugated anti-mouse IgG Fc Ab or HRP-conjugated anti-human IgG Fc Ab for detecting the level of active and inactive pro-Canakinumab by Western blot. Pro-C HC means heavy chain of pro-Canakinumab.


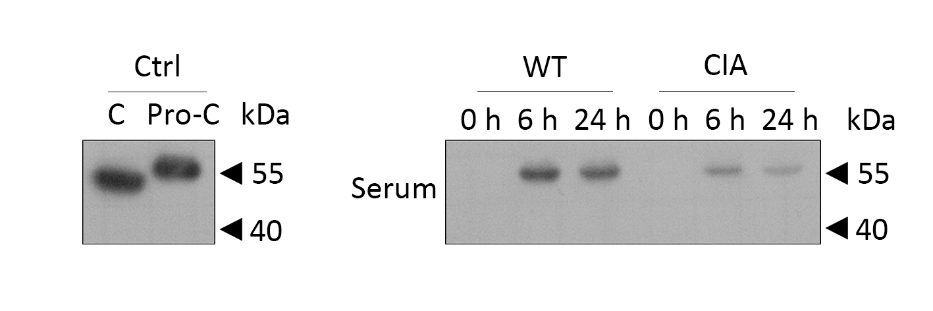


**Supplementary Figure S3.** We treated 50 μg pro-Canakinumab to WT mice (DBA/1 mice) or CIA mice model, respectively, collected their blood samples (serum) at different time points (0, 6 and 24 h after treatment) and using HRP-conjugated anti-human IgG Fc Ab for detecting the level of active and inactive pro-Canakinumab by Western blot. C means Canakinumab. Pro-C means pro-Canakinumab. Ctrl means control. CIA means collagen-induced arthritis.

**Original SDS-PAGE version of Supplementary Figure 2**

**
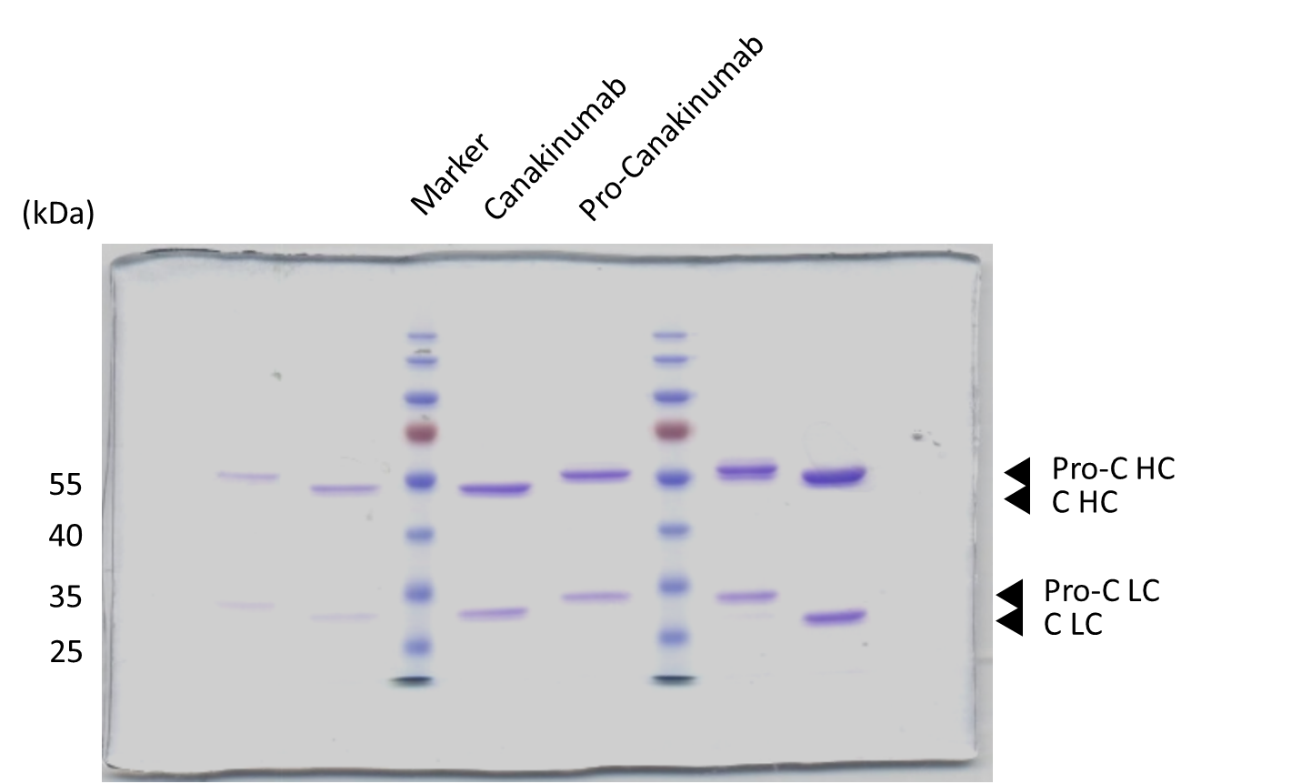
**

**Original blot version of Supplementary Figure 3**

**
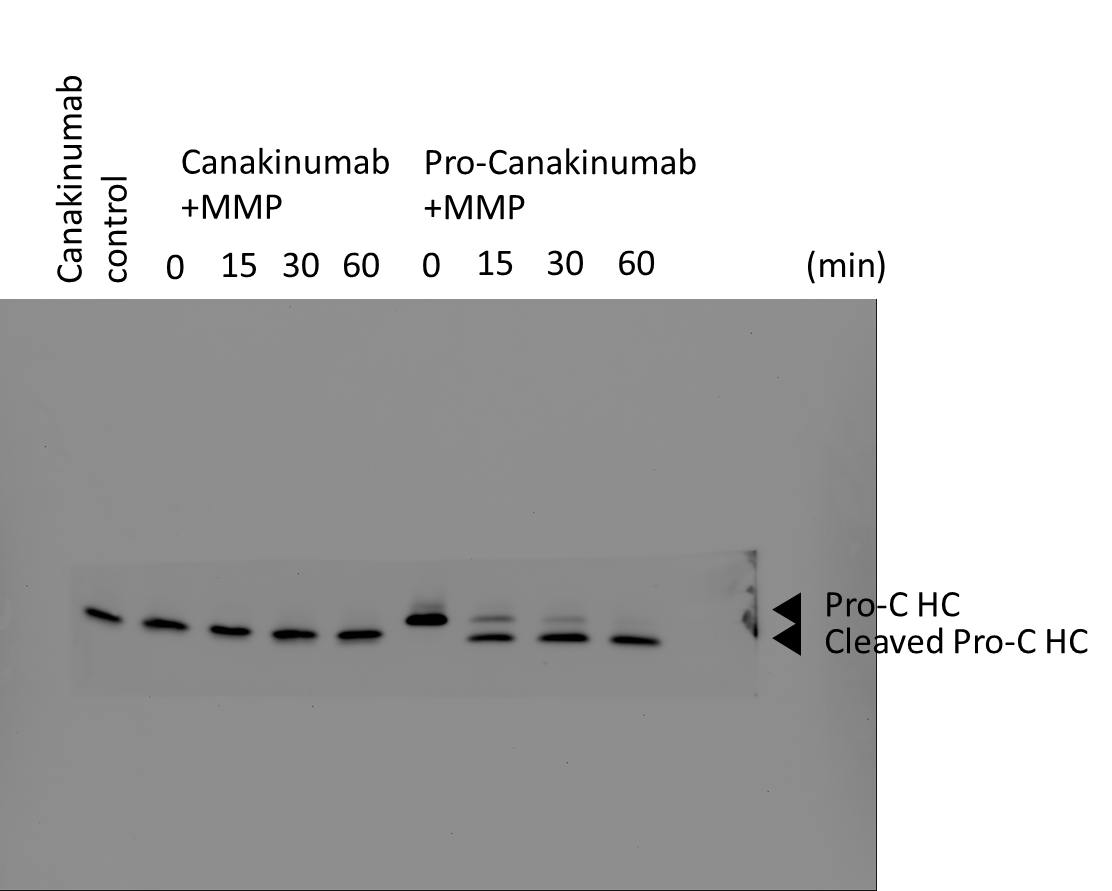
**

**Original blot version of Supplementary Figure S2**

**
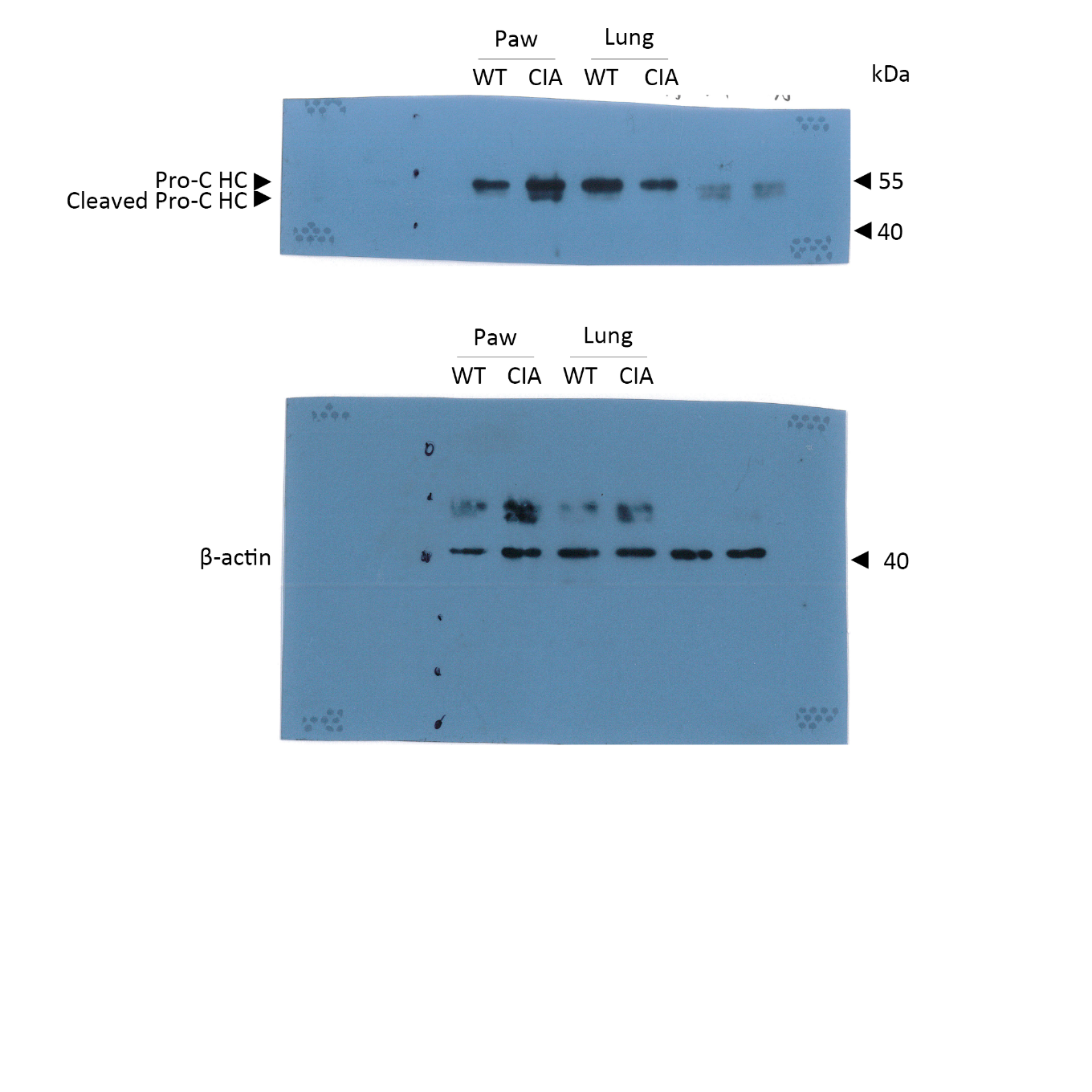
**

**Original blot version of Supplementary Figure S3**

**
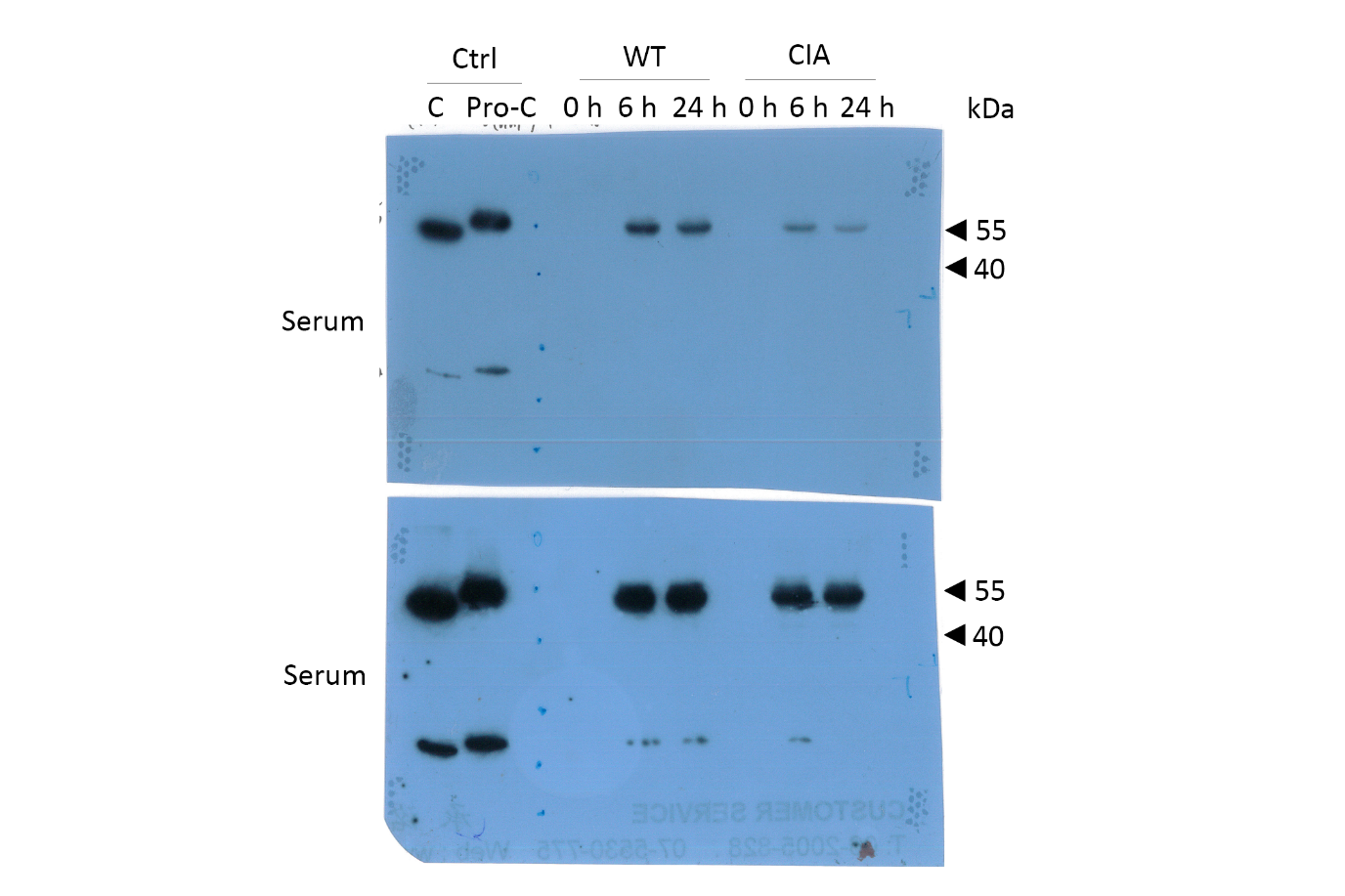
**

**References**

1. Brand, D. D.; Latham, K. A.; Rosloniec, E. F. Collagen-induced arthritis. *Nat Protoc* **2007,** 2 (5), 1269-75 DOI: 10.1038/nprot.2007.173.
